# The relationship between estradiol-progesterone alterations after ovulation trigger and treatment success in intrauterine insemination cycles

**DOI:** 10.4274/tjod.45656

**Published:** 2016-06-15

**Authors:** Tayfun Kutlu, Enis Özkaya, İlhan Şanverdi, Belgin Devranoğlu, Cansu İpekçi, Birsen Konukçu, Yavuz Şahin, Ateş Karateke

**Affiliations:** 1 Zeynep Kamil Women and Children’s Health Training and Research Hospital, Clinic of Obstetrics and Gynecology, İstanbul, Turkey

**Keywords:** Estradiol, progesterone, intrauterine insemination, ovulation induction

## Abstract

**Objective:**

To assess the relationship between the estrogen-progesterone alterations before and after ovulation trigger and treatment success in intrauterine insemination (IUI) cycles.

**Materials and Methods:**

Two hundred fifty-one women with infertility underwent ovulation induction followed by IUI. For all subjects, estradiol and progesterone concentrations were evaluated on the trigger and IUI day. The results were analyzed to assess the relationship between hormone levels and positive pregnancy test.

**Results:**

There were 34 women with a positive pregnancy test following controlled ovarian stimulation and IUI cycle. Estradiol and progesterone levels on the trigger day and the day of IUI were compared within groups with and without positive pregnancy tests. The comparison revealed significantly increased levels of progesterone after trigger in both groups; however, although there were estradiol level drops in both groups, the drop in the group with negative pregnancy tests was statistically significant.

**Conclusion:**

Significant drops in estradiol concentrations after ovulation trigger are associated with IUI cycle treatment failure.

## PRECIS:

Significant drops in estradiol concentrations after ovulation trigger are associated with intrauterine insemination cycle treatment failure.

## INTRODUCTION

Monofollicular development should be the main goal in intrauterine insemination (IUI) cycles. Controlled ovarian stimulation (COH) was found associated with supraphysiologic estradiol levels and might affect endometrial implantation. Also, some data showed increased success rates with high peak estradiol levels, which were presumed to be indirect evidence for oocyte quality, whereas some studies showed poorer outcomes due to the detrimental effect of high estrogen on endometrial receptivity^(1,2,3,4)^. Although we do not expect to observe estradiol in concentrations so high in IUI cycles that they would be detrimental to endometrial receptivity, it may be used as a reflection of oocyte quality. Progesterone is thought to be the dominant hormone during the luteal phase of the cycle and the endometrial window of implantation is mainly regulated by progesterone and progesterone- induced gene regulations; this effect is strictly regulated. Data showed that increased mid luteal serum progesterone levels were not associated with a higher clinical pregnancy rate in women who underwent COH with IUI. However, a lower mid luteal progesterone level was proposed to be a predictor for treatment failure^(5)^. Other data from in vitro fertilization (IVF) cycles showed a detrimental effect of increased progesterone concentrations (>2.0 ng/mL) before ovulatiovn trigger on oocyte quality and therefore embryo quality^(6)^. In another study, a 10% reduction in estradiol concentrations after ovulation trigger was associated with 40-50% lower clinical pregnancy and live birth rates in IVF cycles^(7)^. Although serum estradiol concentrations are one of the main parameters in the assessment of the response to controlled ovarian stimulation, the predictive value of estradiol levels before or after ovulation trigger is still not known. Some data showed a poor predictive value of serum estradiol concentration alone on the day of recombinant-HCG in IVF outcomes^(8)^. A recently published study proposed the use of the post-recombinant-human chorionic gonadotropin (HCG) estradiol level as an additional component to predict the outcome of an IVF cycle just before oocyte pick-up. The authors indicated the necessity for further studies to clarify the underlying mechanisms that might result in a decrease in postrecombinant-HCG estradiol levels, so that physicians may be able modify following IVF cycles accordingly^(7)^. The aim of this study was to assess the relationship between estrogen-progesterone alterations after the ovulation trigger and treatment success in IUI cycles.

## MATERIALS AND METHODS

In this cross-sectional study, we included 251 IUI cycles performed in the infertility clinic of Zeynep Kamil Women and Children’s Health Training and Research Hospital between 2012 and 2014. This study was approved by the Institutional Review Board of the Zeynep Kamil Women and Children’s Health Training and Research Hospital. All participants gave signed informed consent. All couples had attempted to conceive for at least one year prior to undergoing COH+IUIs. A self-administered questionnaire was used to collect data about demographic, menstrual, and obstetric characteristics. The study population comprised all couples who were candidates for COH+IUI. Indications for IUI included subfertile male infertility, polycystic ovary syndrome, mild or minimal endometriosis or unexplained infertility and various ovulatory disorders. Subfertile male infertility was defined as per the criteria outlined by Molinaro et al.^(9)^. The Initial evaluation included the cycle’s day 3 hormone profile, and tubal patency as determined using hysterosalpingogram and/or laparoscopy. Exclusion criteria were hydrosalpinx, anatomic abnormalities, infection, and systemic disease before intervention.

Ovarian Stimulation Protocol

Transvaginal ultrasonography was performed for each participant on day 3 of the menstrual cycle and daily 75-100 IU recombinant FSH (Gonal-F; Merck Serono, İstanbul, Turkey; and Puregon; MSD, İstanbul, Turkey) injection was started. The ovarian response and endometrial thickness was started to be assessed by transvaginal ultrasound starting from the 5^th^ day of stimulation. If the leading follicle’s diameter was  <10 mm on the 8^th^ day of stimulation, the dose of gonadotropin was increased by 50%. The gonadotropin dose remained the same until the day of recombinant-HCG trigger after the leading follicle reached 12 mm in diameter. Cycles were triggered with 250 μcg recombinant-HCG (Ovitrelle; Merck Serono, İstanbul, Turkey) when the dominant follicle became 18 mm in diameter. Cycles were cancelled if there were more than three dominant follicles and/or estradiol levels >1500 pg/mL to prevent ovarian hyperstimulation syndrome and multiple pregnancies. IUI was performed 36 h after recombinant-HCG administration with a disposable IUI catheter (Embryon; Rocket Medical, Washington, Tyne and Wear, U.K.) by two of the authors. The patient was recommended to rest in a supine position for 15 min after the procedure. Luteal phase progesterone support was started following insemination and continued until a pregnancy test was performed. Luteal phase progesterone support was administered in the form of 90 mg (8%) vaginal gel (Crinone, Merck Serono; İstanbul, Turkey). β-HCG was tested on the 15th day of the post insemination day sample. Luteal phase support was continued until 12 weeks of gestation.

## RESULTS

**Figure 1:**
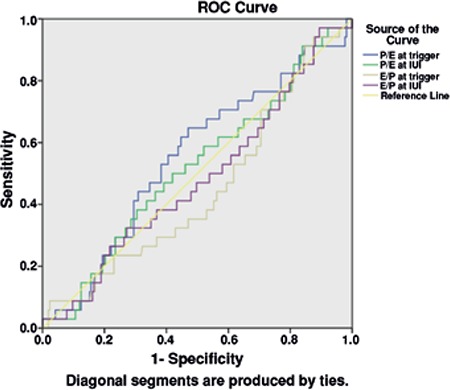
Receiver operating characteristic curve of different ratios to predict positive pregnancy test ROC: Receiver operating characteristic, IUI: Intrauterine insemination

There were 34 (13.5%) women with a positive pregnancy test following controlled ovarian stimulation and IUI cycle. Estradiol and progesterone levels on the trigger day and the day of IUI were compared within groups with and without positive pregnancy test. The comparison revealed significantly increased levels of progesterone after trigger in both groups; however, although there were drops in estradiol levels, the drop in the group with a negative pregnancy test was statistically significant (Tables 1 and 2). The groups were compared in terms of some demographic and hormonal concentrations, the results of which are summarized in Table 3. Estradiol/progesterone at trigger, estradiol/progesterone at IUI, progesterone/estradiol at trigger, and progesterone/estradiol at IUI, all these ratios failed to predict treatment success (p>0.05, Figure 1).

## DISCUSSION

In this study, we assessed the effect of estrogen and progesterone alterations before and after ovulation trigger on IUI cycle outcomes. Our data revealed that a significant drop in estradiol levels after ovulation trigger leads to unfavorable results in IUI cycles. A progesterone rise was not found to have a significant impact on cycle outcome and progesterone levels on trigger day were not significant predictors of cycle outcome. According to our literature search, although there are some data for IVF/intracytoplasmic sperm injection (ICSI) cycles, hormonal alterations during the periovulatory period were not investigated in IUI cycles in detail.

Consistent with our result, previous study on 1712 IVF cycles revealed similar results and indicated estradiol drop >10% after trigger was associated with lower pregnancy rates^(7)^. The mean estradiol drop was 21% in the group with negative implantation and was 11% in successful cycles in our study population.

A study showed a significant association between serum estradiol level on trigger day with the pregnancy rates following ovarian stimulation and intrauterine insemination^(10)^. Our data analyses revealed no relationship between the estradiol level on the trigger day and pregnancy rates.

Endometrial thickness measurement is the most commonly used parameter to have an indirect idea about endometrial receptivity; an optimal endometrial thickness is required for favorable outcome. However, the use of endometrial thickness alone was found to have high negative predictive value but low positive predictive value with low specificity^(11)^. It is well known that endometrial development requires the combined effect of estrogen and progesterone. This combination effect should be in balance, previous data showed significant predictive value of progesterone/estradiol ratio at the periovulatory phase in estimating the efficacy of the ovulation induction in IUI cycles^(12)^; however, our data showed no association between the progesterone/estradiol ratio either at the trigger or on the day of IUI, which indicates that endometrial receptivity is not under a dominant effect of any of these hormones.

Estradiol supplementation has been used to ameliorate endometrial receptivity in IUI cycles. A study on this issue assessed the effect of estradiol supplementation in cycles with luteal phase serum estradiol drop by more than 50% over a 48-hour period within 10 days of recombinant-HCG administration, and the data showed that estradiol supplementation resulted in higher rates of pregnancy (12.6 vs. 20.9%); the difference was more prominent when data were analyzed for patients aged >35 years^(13)^. Consistent with our results, that study also showed a critical role of estradiol during luteal phase. In our study population, ovulation was triggered by recombinant-HCG in all patients. There is evidence about the effect of recombinant-HCG on ovarian endocrine function, a study showed that higher doses of recombinant-HCG administration promoted the secretion of both estradiol and androgens into the follicular fluid, with a shift toward a more androgenic milieu^(14)^. This shows the ovarian endocrine response to recombinant-HCG exposure, which indicates a formation of androgenic state in the ovary. Accordingly, one would expect to observe increased serum sex hormone levels after ovulation trigger; however, our data showed decreased estradiol levels both in the pregnant and non-pregnancy groups, and higher decrements resulted in failure of IUI cycle.

Besides the effect of estrogen on the endometrial receptivity, a premature increase in progesterone concentrations in stimulated cycles was found to have a negative impact on pregnancy rates. Although the exact cause of this progesterone concentration elevation is not clear, it was suggested that overstimulation may lead to increased progesterone concentrations at the end of the follicular phase. Furthermore, this premature progesterone elevation was associated with altered gene expression and also reduced endometrial receptivity^(15)^. In our study, we did not see a significant predictive value of progesterone on the trigger day, the mean values were comparable between the groups (0.8 vs. 1.2, p>0.05). A recent study suggested freezing all embryos in IVF/ICSI cycles if the progesterone level was >1.5 on the trigger day^(16)^. A modest elevation of progesterone was observed in our study, this was thought to be due to the mild stimulation protocols specific for COH+IUI cycle.

Previous study analyzed the additive value of progesterone level determination 24 hours after recombinant-HCG administration and revealed an improved predictive value compared with a single measurement on the day of recombinant-HCG administration, the authors concluded that the high progesterone levels on both days resulted in low implantation rates compared with normal levels in IVF/ICSI cycles (22% vs. 36%)^(17)^. There is also some evidence that basal progesterone levels may be used to predict premature progesterone elevation in IVF/ICSI cycles^(18)^. In our data, post trigger progesterone levels obtained from the laboratory analyses at 36 hours after trigger were not found to affect cycle outcome.

The estradiol/progesterone ratio on the day of embryo transfer has been used to predict implantation in ICSI cycles. A study on this issue indicated that this ratio was predictive for ICSI success when combined with embryo quality, endometrial thickness, and estradiol levels, and higher ratios were found associated with favorable results^(19)^. This study emphasized the role of estrogen during the luteal phase of the cycle stressing that higher ratios were found to be predictive for desirable outcome. Progesterone plays an important role during the luteal phase for decidualization changes and progression of pregnancy. Premature progesterone elevation is observed in 5 to 30% of treatments despite the use of GnRH analogs in assisted reproduction technique (ART) cycles^(20,21,22)^. Some studies revealed favorable outcomes in cycles with elevated progesterone/estradiol ratios with higher oocyte collection and normal pregnancy rates^(20,23,24)^. In contrast, other data showed low ovarian reserve and reduced oocyte retrieval in patients with high ratios^(25)^.

There is no consensus at to whether embryos should be transferred in women with a premature rise in progesterone. There are also no data in the literature regarding optimal stimulation protocols to avoid premature progesterone rises.

We know about the detrimental effect of premature rise in progesterone levels on the ART cycles. However, a study reported a significant correlation between increased progesterone and high estradiol levels and no detrimental effect on the cycle outcome^(26)^. In fact, unfavorable effects of stimulation have been proposed to be observed in the early luteal phase of the cycle and these effects were thought to be corrected during the late luteal phase^(27)^. Consistent with this argument, Elgindy et al.^(28)^ documented different implantation rates between cleavage stage embryo transfer and blastocysts transfer. The authors claimed that the adverse effect of the progesterone/estradiol ratio in stimulated cycles was compensated for by a day 5 embryo transfer^(28)^. The authors of a review on the regulation of steroid production and its function within the corpus luteum concluded that oxytocin and prostaglandin F alpha were found to stimulate estradiol and progesterone release and estradiol itself further stimulated progesterone release. Furthermore, it was also reported that during luteolysis, invading macrophages secrete tumor necrosis factor, which inhibits the luteotropic effects of estradiol and disrupts the intraluteal circuit^(29)^. These data partially explain why we experienced lower rates of pregnancy in women with high estradiol drop after ovulation trigger; an average estrogenic effect is necessary for optimal corpus luteum function. The proposed underlying mechanisms of insufficient function of the corpus luteum included “supraphysiologic estradiol level, decreased luteinizing hormone level, inhibition of the corpus luteum, and asynchronization of estradiol and progesterone”^(30,31)^. A Meta-analyses on estrogen plus progesterone replacement during luteal phase of the cycle showed higher rates of clinical pregnancy compared with progesterone alone in women undergoing IVF^(32)^. According to this and data from our study, an average but not supraphysiologic estrogen function seems to be mandatory during the luteal phase of ovulation induction cycles; significant drops in the estradiol concentrations after ovulation trigger are associated with IUI cycle treatment failure.

## Figures and Tables

**Table 1:**
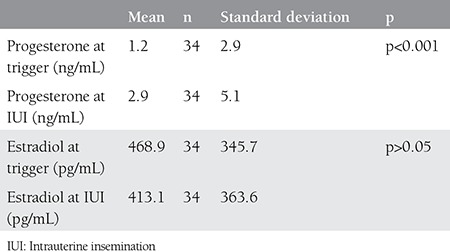
Hormone concentrations on the trigger and intrauterine insemination day in the positive pregnancy test group

**Table 2:**
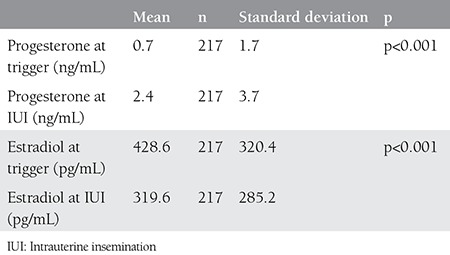
Hormone concentrations on the trigger and intrauterine insemination day in the group with negative pregnancy tests

**Table 3:**
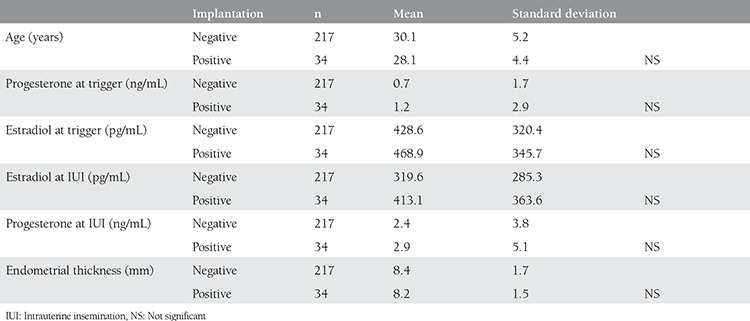
Comparison of groups with and without positive pregnancy test and demographic and hormonal characteristics
